# Role of Muscle LIM Protein in Mechanotransduction Process

**DOI:** 10.3390/ijms23179785

**Published:** 2022-08-29

**Authors:** Philippe Germain, Anthony Delalande, Chantal Pichon

**Affiliations:** 1 UFR Sciences and Techniques, University of Orleans, 45067 Orleans, France; 2 Center for Molecular Biophysics, CNRS Orleans, 45071 Orleans, France; 3 Institut Universitaire de France, 1 Rue Descartes, 75231 Paris, France

**Keywords:** mechanotransduction, striated muscle, ultrasound stimulation, prophylaxis, atrophy, MLP

## Abstract

The induction of protein synthesis is crucial to counteract the deconditioning of neuromuscular system and its atrophy. In the past, hormones and cytokines acting as growth factors involved in the intracellular events of these processes have been identified, while the implications of signaling pathways associated with the anabolism/catabolism ratio in reference to the molecular mechanism of skeletal muscle hypertrophy have been recently identified. Among them, the mechanotransduction resulting from a mechanical stress applied to the cell appears increasingly interesting as a potential pathway for therapeutic intervention. At present, there is an open question regarding the type of stress to apply in order to induce anabolic events or the type of mechanical strain with respect to the possible mechanosensing and mechanotransduction processes involved in muscle cells protein synthesis. This review is focused on the muscle LIM protein (MLP), a structural and mechanosensing protein with a LIM domain, which is expressed in the sarcomere and costamere of striated muscle cells. It acts as a transcriptional cofactor during cell proliferation after its nuclear translocation during the anabolic process of differentiation and rebuilding. Moreover, we discuss the possible opportunity of stimulating this mechanotransduction process to counteract the muscle atrophy induced by anabolic versus catabolic disorders coming from the environment, aging or myopathies.

## 1. Introduction

Specific conditions, pathologies or aging can cause muscle atrophy. The decrease in strength, endurance or muscle mass and its effect on cardiovascular or locomotor systems may induce a spiral of deconditioning that results in the loss of several physiological capacities [1]. In 2045, it is estimated that at least 64% of the European population will be impacted with sarcopenia or cachexia due to aging and chronic hypoactivity, which could be a major public health issue [2,3]. In 2016, the NIH has already started to determine which type of prophylaxis could be proposed to counteract this issue in a more global manner.

At present, there is a strong connection between physical activities and good health as part of an integrative approach for health and wellness [4,5]. In this context, the mechanotransduction process should not be defined only as a mechanical stimulus transduction into an electrical signal as described in the 1980s [6,7], but also as the mechanism through which physical forces are converted to biochemical signaling pathways inside the cells. Therefore, it is crucial to explain the relationship between physical stress and physiological adaptation [8]. This review is focused on how a strain applied to the striated muscle protein content [6] can result in an adaptive response [7] and, particularly, on the molecular mechanisms of skeletal muscle hypertrophy [8] through the stimulation of specific mechanosensors [9,10]. 

The focus of this review will be on the muscle LIM protein (MLP, also known as cysteine and glycine rich protein 3 (CSRP3)), which is a mechanosensitive protein. Moreover, we will provide a description of its role during the activation process. 

## 2. The Plasticity of Muscle

The loss of neuromuscular system functional capacity can be related to an alteration in muscle plasticity. This can occur in skeletal, cardiac or striated muscles that undergo atrophy, especially in a context of a lack of mechanical stress [11]. In contrast, muscle hypertrophy could occur in response to exercises or pathology as hypertrophic cardiomyopathy [12,13]. These events are consequences of the modification of biochemical contents that appear upon alteration in the innervation or the endocrine system as well as physical activity [14]. 

Adaptive mechanism is described by the adaptive homeostasis [15] and the compensation induced by the hormetic dose-response (sublethal damage caused by small doses of a toxin or poison and an exaggerated repair response) [16,17] relative to the proteins turnover cycle [18]. Contrary to overcompensation, aging or some pathologies and the environment are the principal factors of muscle deconditioning. For example, when the environment is involved in deconditioning, the consequence is a lack of activity, hypokinesia or hypodynamia. Several experimental models, such as immersion [19] or bed rest [19] for humans and rat hindlimb suspension [20] have been used and the structural, functional, kinetics, and amplitude of the adaptations have been characterized. The muscle force decrease in reference to the speed of contraction [21] and the rise of fatigability [22] has been quantified. Changes have been primarily explained by a global neuromuscular degeneration [23], atrophy or slow-to-fast fiber switching [20], sarcomeric or costameric modifications [21,23,24], overproduction of no contractile proteins, such as collagen leading to fibrosis [25], and occasionally apoptosis [26]. Vernikos and Schneider reported that exposure to microgravity environments can induce neuromuscular atrophy compared to age-induced atrophy, albeit with a possible reversibility [2]. In contrast, sarcopenia which is the muscle atrophy caused by ageing, is hard to counteract [27,28]. Moreover, atrophy may directly result from pathological conditions, for instance, as observed in Duchene’s muscular dystrophy [29,30] or it can be a consequence of, for example, the multifactorial pathology and often irreversible wasting syndrome, cachexia, that is well known to occur during cancer [31].

Atrophy versus hypertrophy (the opposite process) could occur through an adaptive mechanism, which is caused by several types of environmental stimuli translated in the activation of specific signaling pathways. Many researchers have investigated the hypertrophy and atrophy of striated muscle fibers as cardiomyocytes [13,32,33,34] and rhabdomyocytes [35,36,37], enlightening the relationship between the adaptive biochemical strategy of the cell and the specificity of the homeostatic imbalance. Therefore, myocyte atrophy and hypertrophy are considered as the result of this imbalance [38].

For striated muscle hypertrophy, several pathways imply specific triggers, ligands or receptors before the induction of different cascades of events. The trigger signals may be soluble molecules coming from endocrine secretions, such as adrenaline/PKA [39], insulin/PI3K [40] and thyroid hormones/nuclear thyroid receptors [41] or cytokines, such as cardiotrophin/SHP2 [42], LIF/Ras [43], and TGF/TAK1 [44]. Other signals involve amino acids, such as leucine/mTOR [45], microRNAs [46] or nitrogen oxide [47].

At first, it was thought that the endocrine system played the only role in muscle hypertrophy. To date, signaling pathways from membrane proteins can sense extracellular mechanical stress, which appear to be increasingly important. When a mechanical stress is applied to the plasma membrane, the cell has two possible major responses: (i) Modification of the cell permeability through calcium channels, such as TRPCs [48,49] and (ii) transmission of this mechanical stress [50,51,52] through a pathway involving different signaling molecules, including Src, FAK, ILK, and MAPKs/MEKK1. Apart from these molecules, biomechanical sensors and their stimulation (or lack of thereof) also lead to muscle hypertrophy and atrophy, respectively [53]. The term sensor can be defined as any structure that is able to induce a deformation under mechanical stress and provide information on post-translational modification.

In the following section, we will focus on stress perception through mechanobiological models and theories [54,55,56] as well as the incidence of stress on the heart [57] or musculoskeletal system [12].

## 3. Mechanical Stress and Mechanotransduction

As reported by Rindom and Vissing, the field of mechanobiology investigates how the mechanical stress applied to the cell is detected and the immediate or delayed effects of this stress on extracellular or cellular responses [6,51,58,59].

### 3.1. Mechanobiology versus Biomechanics

Several studies have investigated the field of mechanobiology since it links the mechanical stimulation of the whole cell or the extracellular matrix components to the mechanical or biochemical response of the cellular ultrastructure [58]. Under external mechanical stress, the cell responds through an adaptation of signaling pathways that result in boosting or building specific mechanisms [59]. In contrast, the notion of biomechanics refers to the mechanical response of cells and tissues to the stress (stiffness, strength, time to response). Consequently, the biomechanics are often associated with the macrostructural level [60] and sometimes associated with the ultrastructural level [61]. Therefore, for more insight into the mechanobiological process, it is crucial to define how the mechanical stress is transmitted through the extracellular and intracellular matrices (ECM/ICM).

### 3.2. The Mechanical Stress

Stress can be defined at the level of both cell and tissue [62,63]. Therefore, it is possible to investigate the mechanical effects from a macrostructural scale to an ultrastructural scale [7,64]. Forces can be applied to the whole organ in several ways, such as compression, traction, torsion, flexion or shearing. The ECM, in turn, transmits these forces to the transmembrane molecular complexes before generating a tension of cytoskeletal proteins and, eventually, leading to a sensor activation and biochemical transduction. This allows for the understanding of why stress production is able to induce cellular adaptations and explains the choice of the term mechanotransduction despite its original meaning [6,7]. In this context, physical activity will be a high-level mechanical stress generator through musculo-tendinous tensions, articular compressions or flexion of bone diaphysis. The mechanotransduction induced by exercise can induce muscle protein synthesis leading to hypertrophy, as proposed by Rindom and Vissing [51] and demonstrated by Petriz et al. [65].

At the macrostructural level, we need to consider the impact of a global stimulus (for instance, adaptations to exercise training) on the mechanotransduction process. Our previous findings [66] show, for example, the positive incidence of mechanical impacts induced by intermittent treadmill exercises at high intensity on the activation of a signaling pathway involving FAK and FHL2/KLF8 dependent pathway, which is involved in the differentiation of cells from bone marrow in the radius bone of the rat.

At the microstructural level, for example, Hua et al. (2016) have demonstrated that in vitro mechanical stretching (10% deformation at 0.125 Hz) of murine C2C12 myoblasts resulted in their differentiation and proliferation [67] through the NF-κB signaling pathway. Juffer et al. have compared two types of shear stress (by laminar flux or axial cyclic deformations) exerted on the myofiber extracellular matrix and have concluded the important role of shear stress on mechanotransduction in the muscle with an increased expression in IGF-I Ea, MGF, VEGF, IL-6, and COX-2 genes, and a decreased expression in myostatin gene [68].

Tsimbouri et al. have proposed the concept of nanostimulation at the ultrastructural level with regard to the impact of a mechanical stress at the nanometer scale on molecular complexes [69]. The impact of *nanoscale manipulation* has been studied using silica nanopillars to induce 50 to 500 nm of membrane curvature. As a result, it was found that clathrin and dynamin 2 proteins colonize with the nanopillars, suggesting that these nanostructures could trigger clathrin-mediated endocytosis in live cells [70]. The mechanosensing by cytoskeletal proteins and the activation of molecular mechanism have been reviewed by Hu et al. [71]. As an example, the application of low intensity pulsed ultrasound (LIPUS) at 3 MHz on chondrocytes triggered a specific stimulation of focal adhesions through integrin complexes of the initiation of a signaling pathway leading to FAK-PI3K/Akt mechanochemical transduction [72]. 

To process all the data obtained at the macro-, micro-, and ultrastructural levels, modeling studies of the mechanical stress incidences on biological systems have been performed. Mittag et al. [73] have used mathematical simulations to investigate whether the form of the diaphysis is a result of mechanical strain. In this case, the simulation was based on a mechanistic approach for mechanotransduction and bone formation. Concerning this mechanistic approach, Hughes et al. reported that during the sliding of sarcomeric proteins in muscle cells in opposition to an extracellular mechanic resistance, there is a longitudinal chain of forces transmission and a lateral chain of forces transmission between the extracellular and intracellular matrices [63]. The longitudinal forces inside the ICM are associated with the stress coming from external forces or sarcomeres shortening. However, in addition to this longitudinal force transmission, a lateral force transmission through the endomysium is another form of ICM solicitation [74]. This lateral transmission corresponds to a stress applied perpendicularly to the cell plasma membrane and, consequently, ICM proteins stretch can be induced by a membrane action/reaction phenomenon. Therefore, when a pressure is applied on the cell plasma membrane, it induces first the formation of a curvature, then a reactive depression occurs at the end of this pressure, resulting in the formation of an opposite membrane curvature. Any stimulation by a pressure is associated with a compression/stretching cycle of ECM and ICM macromolecules. The membrane curvature becomes a feature of the cell membrane, defined by the cytoskeletal organization. This nanoscale modification of membrane curvature (in the range of 100–200 nm) may constitute one mechanism of communication between intra- and extracellular matrices as well as the mechanism of stimulation [75]. 

Ultrasound is defined as a mechanical wave created by an alternation of positive and negative pressure phases at a frequency over 20 kHz. When ultrasound waves are applied as mechanical stimuli on the cells, the sound pressure is also able to induce positive or negative membrane curvatures and, consequently, the pressure and the stretch of transmembrane and subsarcolemal proteins. As an outcome, the stiffness of the cytosolic protein organization and the stress applied on these proteins could induce a form of tensegrity, involving pillars and ropes that result in a possible signaling pathway by mechanotransduction. For instance, ultrasound-based-therapy is used by a physiotherapist to enhance the tendon or cartilage healing process. It is hard to dissociate the contribution of thermal and mechanical effects involved in the healing phenomenon and it is possibly a combination of both. Sonoporation (also known as an ultrasound contrast agent) is one technique based on microbubbles activation by an ultrasound for plasma membrane permeabilization, which allows for drug or gene intracellular delivery (see Delalande et al. [76] for review). At the cellular level, and as a consequence of plasma membrane modification, an ultrasound can induce calcium uptake, formation of actin stress fibers, endocytosis or chromatin reorganization. 

**Figure 1:**
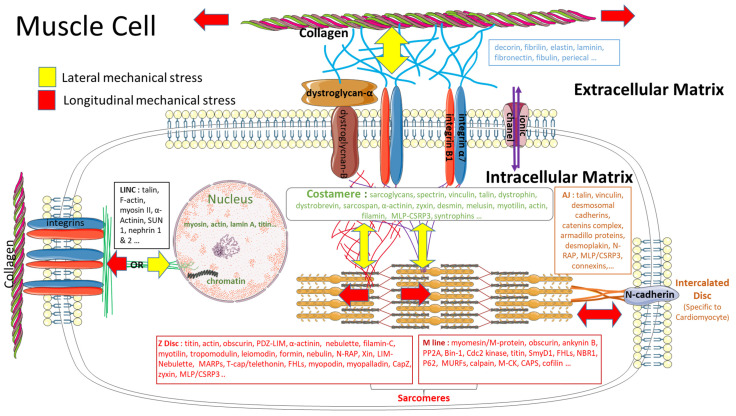
Relationship between the costamere and the sarcolemma, the extracellular matrix and the sarcolemma and, the sarcolemma and the nucleus. The drawings of costamere zone, extracellular zone and the nucleus zone are inspired from Samarel et al. [100], Nishimura [77], Wang et al. [75] and Henderson et al. [101], respectively. Red arrows: longitudinal force transmission. Yellow arrows: Lateral force transmission. An example is shown on the possible mechanical stress induced by an external pressure or internal sarcomeric contraction.

Figure 1 is a representative scheme that summarizes the molecular structures and forces that will take place when a mechanical stress is applied. It shows the relationship between the costamere and sarcolemma, the extracellular matrix and sarcolemma, and the sarcolemma and nucleus, as well the different proteins involved in the transmission of forces from the outside into the inside of the striated muscle cell up to the nucleus. Herein, we will describe the structural organization of ECM components and the interactions between ECM and ICM through integrin and dystroglycan complexes (including the costameric zone), as well as the physical link between the integrin and nucleus.

### 3.3. Impact of a Mechanical Stress on the Extracellular Matrix

During muscle contraction or muscle stretching, proteins of the intramuscular conjunctive tissue (IMCT) will be used for the force transmission and despite some anatomical differences, such as length, focal, and/or adherent junction, cardiac and skeletal cells have a number of similarities, which allow us to build a common model of mechanical constraints chain, as shown in Figure 1 [77].

When a mechanical stimulus is applied from the ECM, it will be communicated to the ICM through the plasma membrane [78]. This communication, which is defined as the cross-talk between ECM and ICM, implies different multiple transmembrane proteins. Ionic channels, integrin complex (ITG), and dystrophin-glycoprotein complex (DGC) are three potential candidates that could play a role in mechanosensors. The mechanical stress coming from the ECM can be sensed through ionic channels, such as NAV1.5, TREK1, KATP, SAKCA, CAV1.2, CFTR, TRCP, TRPV or Piezo1 and Piezo2, which are able to translate the stress into an intracellular ionic flux (see Takahashi et al. [48] or Liu et al. [79] for reviews). One example reported by Pool et al. [80] is the mechano-electro-transduction coming from transmembrane proteins linked to laminin. They are able to transduce a traction into an opening of ionic channels, resulting in intracellular electric events. 

Ion channels are activated by several mechanical forces [81,82]. Piezo1 channel [79] enables the cells to sense radial pressure, membrane stretching, compression, shear stress, matrix stiffness, ultrasound, matrix nano topology, and osmotic pressure. It also allows Ca^2+^, K^+^, and Na^+^ to flow inside the cells when channels are open under a mechanical stress. In this case, a disruption of the continuum of the force transmission can be considered. 

With regard to DGC and ITG, the proteins link the ECM to the subsarcolemma compartments, and once stimulated, they are able to conserve the continuum of force transmission [30]. When connected to the Z-discs of sarcomeres, these proteins are classified as “costameric proteins” [81,83]. The other proteins that are linked to the M line of sarcomere [82] or nucleus [75] have no special name. ITG and DGC are comparable since they both play a structural and mechanical role in the plasma membrane, and they induce responses themselves. Sun et al. [84] have compared the integrins to a molecular clutch involved in a rapid mechanical response and in a deferred response by the gene expression. During a mechanical stress applied to the cardiomyocytes of mice, DGCs are involved in anabolic signaling pathways [85] and the lack of dystrophin (a component of DGC) is quite dramatic. Several studies have reported the involvement of these two protein complexes in myopathies as Duchenne’s muscular dystrophy (see McNally and Peter Pytel [86] or Dowling et al. [87] for reviews).

### 3.4. Impact of a Mechanical Stimulation on the Intracellular Matrix 

The first hypothesis on the Ca^2+^ sensitivity of the cardiac contractile machinery, that may be controlled at least partially by an internal passive load on the titin, has been proposed in 1999 [88]. Later, Knöll et al. [89] and Bos et al. [90] have associated the dysfunction of the “muscle LIM protein” (MLP) of the Z-disc associated with the telethonin (also called T-Cap) to a cardiomyopathy and the mechanic sensitivity of the heart muscle to stretching. Since then, Galkin et al. [91] have implied that actin could act as a mechanosensor. We paid special attention to the work by Kruger and Köter [92], who proposed an hypothesis on the role of the proteins associated with the titin in the elastic sarcomeric band “I”. Their biophysical approach is similar to Cazorla’s work, which proposes the particular importance of the cellular space between the Z-disc of sarcomere and sarcolemma (Figure 1). This zone denominated as “costamere” [81], is crucial for forces transmission and mechanical signaling pathways involved in muscle pathologies, such as myopathies or in muscular cell remodeling [24,83,93].

Recently, the sarcomere “M” line appears as complementary to the Z-line and it is able to transmit forces from the molecular motor to the membrane and the extracellular matrix [94]. Therefore, tropomodulin could act as a mechanosensor with an implication similar to the regulation of the cytoarchitecture of striated muscle cells [95]. Between the plasma membrane and the nucleus, the cytoskeletal structure could also be involved in a mechanical transmission. The continuum between the membrane and the nucleus is composed of a variety of proteins that allow for propagation in a few milliseconds of a mechanical wave, which comes from an extracellular matrix to the nucleus. In comparison, the information by diffusion or translocation of proteins inside the cytosol requires 5 to 10 s. Since there is a transmission of external cellular forces to intranuclear proteins as titin or actin, Guilluy and Burridge [96] proposed the concept of a linker of nucleoskeleton and cytoskeleton (LINC), which is comparable to the focal adhesions found in the plasma membrane [97,98]. The role of titin as a stress sensor in cytoskeleton and its direct mechanical activation in the nucleus is supported by Wang et al. [75]. They showed that a stress wave coming from integrins could be propagated by talin, vinculin, myosin, F-actin, alpha-actinin, and nesprin1 or nesprin2 to the nuclear titin through SUN1 and laminin-A nuclear envelope (Figure 1). 

Interestingly, the role of titin at the sarcomere “M” line in the mechanotransduction process has been evidenced by its interactions with different mechanosensors proteins, including MLP [99]. Full or partial deletion of the titin gene in mice led to a skeletal muscle atrophy with reduced strength and severe sarcomere disassembly. MLP was overexpressed in those mice compared to the wild type.

In summary, the importance of cell sensing is validated by a substantial body of literature that has identified complex molecular networks at various levels from the outside of the ECM level, at the plasma membrane, and inside the cell. At each level, there is an integrated network that plays a role in processing the sensing activity, which allows the cell to respond correctly depending on the physical environment. In the next section, we will review how the cells will correctly translate this sensing to a specific mechanotransduction process, leading to an adaptation of their behavior regarding the environment.

## 4. From Mechanosensitivity to Mechanotransduction

The cell mechanosensitivity related to the mechanical properties of the environment (rigidity, geometry) defines the form, the orientation, the proliferation, the differentiation, the death of the cell, as well as the development and organization of the tissues under specific conditions [50]. For instance, muscle cells are able to respond to strength, pressure, and shear gravity applied through a new reorganization of the cytoskeleton and/or neosynthesis of proteins that will be translated to specific mechanical features (elasticity, rigidity, force, etc.) [61,102]. Therefore, muscle cells respond to external forces by an adaptation. This mechanosentivity is able to initiate the translation of a mechanical stress to an activation of the mechanotransduction process [103]. Considered together, mechanosensitivity and mechanotransduction can not only be regulators of the homeostasis equilibrium and development [104], but also of several responses to the disease (see the nature biomedical engineering editorial “Pathological mechanosensing” issue [105]).

It is crucial to model the behavior of cell responses, while understanding the relationship between mechanosensitivity and mechanotransduction. During the stress, one could ask whether the whole membrane is involved and what is its relation with the cytoskeleton? According to Ingber et al. [7], the elastic stress of the cytoskeleton defines its tensegrity. Several rigid components and pillars are associated with ropes comprising intracellular microtubules, intermediary filaments, and microfilaments. Pillars and ropes as a whole will react to the external stress inducing a mechanical link between the cytoskeleton and ECM [106]. Herein, the pillars correspond to the microtubule and the ropes are microfilaments and intermediate filaments. The cell deformation is possible since the ropes are elastic and the integrity of cytoskeleton is maintained by the stiffness of pillars in a nanoforce equilibrium.

Integrin or dystroglycan complexes could be pillars that transmit the stress to intracellular proteins, leading to an activation of a mechanotransduction [107]. Acto-myosin interaction can generate intracellular constraints. Generated intracellular forces will result in mechanical effects on sensors in a similar way as those coming from the extracellular matrix [108]. Therefore, mechanosensitive proteins are dependent on both external and internal forces. Among the proteins contributing to the cytoskeletal stiffness, the MLP (one protein of the ropes) is particularly interesting. As part of the zinc-finger proteins family, one of the most abundant protein family involved in the mechanosensitivity [109] and the regulation of transcriptional processes [110], MLP has been reported to have a structural and mechanotransductive function [111,112] and could be compared to zyxin, paxillin, FHL2, etc.

## 5. Muscle LIM Protein

### 5.1. MLP Isoforms

MLP or CSRP3, which is first localized in the striated muscle (skeletal or cardiac), is encoded by the CSRP3 gene [113,114]. In fact, MLP is found in different organs, such as the vascular system [115] or in the central nervous system [116,117].

From an ultrastructural point of view, in the striated muscle, the MLP is associated with several proteins in the intercalated disc, in focal adhesions, in sarcomeres, and in the nucleus. In 2009, Gunkel et al. [118] have evoked the strategic position of MLP since it is associated with different transcription factors as MyoD, myogenin or MRF4 in the nucleus. In addition, it plays a role in the continuum involved in mechanical stress transmission between the ICM and ECM at the costameric or sarcomeric levels (Figure 1).

Rich in cysteine residues, MLP is composed of 194 amino acids with a molecular weight of 21 kDa. It is characterized by two LIM domains that bind zinc and is involved in the interaction between MyoD and spectrin β. This protein bears different zones for anchoring telethonin, alpha-actinin, cofilin-2, N-RAP, actin-F, vinculin, CGMP kinase1, RyR2, NF-κB, and caveolin. Moreover, it bears a nuclear localization signal (64 to 69 amino acids) [29].

At present, two isoforms are distinguished. The first long isoform is denominated as MLP-a and MLP-b, and the second isoform is composed of 59 amino acids, as identified by Vafiadaki et al. [119]. The N-terminal part, which is composed of the first 37 amino acids of those isoforms, are homologous. MLP-b has only one LIM domain and is especially localized in the sarcomere. It is connected to the Z-disc components as alpha-actinin, teletonin, CFL2, and to the MLP-a. 

Initially, MLP (without differentiation of the isoform and likely related to MLP-a) is defined as a regulatory component involved in proliferation or myogenic differentiation. A deficit or a mutation [114] in MLP induced a cyto-architectural disorganization associated with the heart to a dilated cardiopathy, a cardiac failure or an impairment of intercalated disc as well as a dysfunction of passive ventricular activity. In the heart of patients with a deficit in MLP as in dystrophin, there are compensatory mechanisms initiated by the synthesis of desmin or dystrophin [120]. The downregulation of MLP expression can induce a deficit in mitochondrial contents and dysfunctions of coupling excitation-contraction and of the muscle contractility leading to fibrosis, especially for the elderly [121].

***MLP-a*** is primarily expressed in the myoblast during and after differentiation in the myotubes. This protein is involved in the activities of MyoD, MRF4, and myogenin as a cofactor of sarcomeric or costameric proteins. It is also involved in the regulation of intracellular nitrogen oxide, cell differentiation, and integrin-mediated myoblast adhesion. MLP-a plays a role in myo-typological transitions, especially for fast-to-slow fiber interconversions. The overexpression of MLP-a increases its amount in the nucleus, resulting in the fusion of myoblasts with an increase in the ratio length/diameter [122]. 

***MLP-b*** is localized in the cytoplasm during the earliest phase of the myoblast differentiation and is supposed to be involved in sarcomere synthesis and integrity [122]. MLP-b overexpression resulting from an increase in the MLP-b/MLP-a ratio is associated with pathologies, such as the limb-girdle muscular dystrophy type 2 (LGMD2A) or Duchenne muscular dystrophy (DMD). A simple increase in MLP-b reduces the cellular differentiation by its interaction with CFL2, leading to a negative effect on the development of the cytoskeleton and on the myotube fusion by actin depolymerization [122].

MLP-b does not play any role as a transcription factor and MLP-a seems to be the sole isoform associated with the anabolic process. Boateng et al. [123] have investigated the content of MLPs associated with the cytoskeleton, found in the cytosol, at the plasma membrane and in the nucleus of myocytes. They have found monomeric and polymeric forms (di, tri or tetramers). MLPs of costamere and sarcomere are polymeric and are associated with the cytoskeleton or cell molecular motors, whereas the monomeric MLPs are exclusively nuclear. 

The role of the mechanosensitivity feature of MLP related to its carboxyl end has been identified during cardiomyocytes stretching [114,123], an infarction [9,124] or an hypertrophic cardiopathy [125]. These specific conditions lead to a decrease in MLPs in the cytoskeleton and an increase in the nuclear part, leading to an increase in the nuclear versus cytoskeletal ratio (up to a factor of 3) and, consequently, the weakness of the cellular architecture.

Despite the initial work by Arber et al. 1994 and the evidence of MLP importance in the myogenesis after stretching, very few reports have been published concerning MLP involvement in skeletal muscles. In 2015, Vafiadaki et al. [122] have reported the central role of MLP-b in the regulation of protein synthesis for the stabilization of sarcomeric and costameric zones in cardiac and striated muscles. Very recently, Koskinen et al. [126] have identified that MLP proteins are associated with the ankyrin and MuRF (through the organizational role of titin) as a mechanical sensor involved during post-traumatic regeneration. Therefore, according to the theory by Ingber [56], MLP would be a rope’s protein that is mechano-sensitive and able to be depolymerized and activated, allowing for its nuclear import where it plays a role as a cofactor of transcriptional factors implied in hypertrophy. For any stress applied, MLP stimulation could be a direct mechanotransduction pathway. Therefore, MLP appears to have a structural and functional importance. 

Notably, different post-translational modifications of MLP have been identified [9,118,122]. They include acetylation, phosphorylation, sumoylation, glutathionylation or polyubiquitinylation. These modifications could be involved in MLP nuclear import. However, to date, despite bioinformatical predictions (NethPhos 2.0 Server [127] or PhosphoSitePlus Sever [128]), there is little knowledge on the role and impact of these modifications on the MLP intracellular transcriptional message.

MLP has multiple localizations at the costamere, intercalated discs, sarcomere level in Z-discs or M-band [87,118,129,130,131], as shown in Figure 1. MLP can be found as a polymeric or monomeric protein [111]. As a polymer, MLP has a structural activity, while its monomeric form which is able to be imported into the nucleus has a functional biologic activity [122,123].

### 5.2. Nuclear Translocation

The study by Boateng et al. [10] on the repeated stretching of cardiomyocyte during 48 h at 1 Hz has revealed that MLP-a is imported from the cytosol to the nucleus and the importance of tRKYGPK as a NLS sequence. As previously mentioned, the mechanical wave transmission into the cytoskeleton occurs in a few milliseconds and different cascades of events (proteins translocation or chemical diffusion) take place from 5 to 10 s. However, the kinetic of protein translocation from the cytoskeleton to the nucleus and vice versa is not well defined [75].

Furthermore, we have observed the nuclear translocation of MLP by performing a mechanical stimulation of myocytes using low impact pulsed ultrasound (LIPUS) with the following parameters: 10 min of stimulation; 1 MHz of frequency; acoustic pressure of 150 kPa; and 20% of duty cycle (Germain et al. unpublished data). The experiments were performed on myocytes obtained from the differentiation of C2C12 murine myoblasts. The kinetic of MLP translocation from the cytoskeleton to the nucleus and back to the cytoskeleton was assessed by varying the time lapse between the cell stimulation and cell fixation (10 s to 2 h). We found that MLP was localized in the nucleus after 6 to 20 min post-stimulation. In contrast, MLP was localized in the cytoplasm from 30 min post-stimulation. These observations suggest a temporality of the nuclear translocation of MLP and its return back into the cytoskeleton. Despite the fact that these data must be completed with a functional study (ongoing), they were in line with those made by Boateng et al. [10], who used another type of stimulus.

Notably, the MLP nuclear transport can also be triggered by a chemical signal, as very recently reported [132]. The authors evidenced that vitamin C supplementation promoted the differentiation of C2C12 cells and the repair of mouse muscle injury by upregulating the nuclear translocation of MLP, allowing its interaction with MyoD and MyoG transcription factors.

### 5.3. MLP Involvement in the Transcription Process

Upon nuclear import, MLP has been found to interact with muscle transcription factors (MFRs), such as MyoD, MRF4, and myogenin [133]. These proteins are involved in the establishment of the muscular phenotype by the regulation of the proliferation and the activation of specific genes for cell differentiation and sarcomeres assembly [134,135,136]. In mature muscles, MyoD and myogenin are downregulated, whereas MRF4 becomes overexpressed for the terminal phenotype expression [137,138]. During the myoblast differentiation, MyoD allows for the expression of several genes, especially those implied in the synthesis of the contractile apparatus proteins, such as myosin light chain, myosin heavy chain, alpha-actinin, and troponin [139,140,141,142,143]. Moreover, MyoD is involved in the expression of myogenin and cadherin 15 [135] and contributes to its own synthesis [144]. Recently, MyoD has been shown to be a regulator of muscle metabolism [145]. Therefore, MyoD is a master regulator of the muscle function and its development. The LIM domain of MLP is involved in its binding to MyoD, resulting in the formation of MyoD-E complex. MLP acts as a cofactor of MyoD for the transcription of myogenic bHLH proteins. During the muscle hypertrophy, it would be worthwhile to add the mechanotransduction pathway that results from the activation by tensegrity of the MLP sensor in signaling pathways, as reported in some reviews [13,36].

### 5.4. MLP Role in Mechanotransduction Process following Mechanical Stimulation

**Figure 2:**
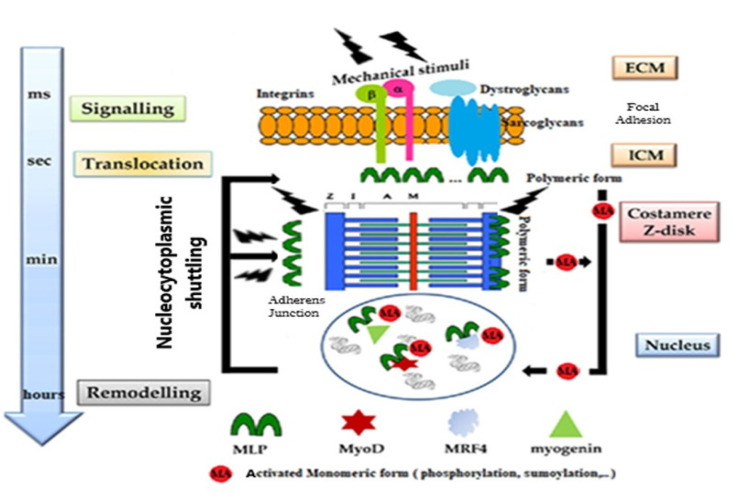
Proposition of events implying the mechanotransduction process induced by MLP following mechanical stimulation. Shown are the different molecular partners that are involved during the cross-talk between the extracellular matrix (ECM) and intracellular matrix (ICM) upon mechanical stimuli. The dual role of MLP as a structural protein in its polymeric form and as a functional protein in its activated monomeric form that plays the role of cofactor of MyoD, MRF4, and myogenin transcription factors. Post-translational modifications as phosphorylation or sumoylation of monomeric MLP triggers its shuttling to the nucleus. Inspired and modified from [9].

Recent studies have highlighted that mechanosensing is highly dependent on the balance between the exogenous and endogenous mechanical stress, corresponding to contractile forces [6,146]. Any physical stimuli that the cells will sense from its environment will be translated into the mechanotransduction process, which comprises biochemical and biological responses. Nevertheless, the cell response is also dependent on the contractile forces present in the intracellular matrix of the cell. The mechanotransduction process is regulated by the cross-talk between ECM and ICM, implying different transmembrane proteins, such as dystroglycan, sarcoglycans or intergrins, for instance [78,147,148]. For the striated muscle cells, the mechanical cross is mainly between ECM and ICM in the skeletal muscle, while in the heart, there is an additional cross-talk between the cells. This involved the adherens junctions AJ and focal adhesions (Figure 2). For other cell types, the cross-talk between ECM and ICM implied in addition to integrins, DGS, and AJ, many other types of complex networks, including podosome, desmosome, tetraspanin-enriched-microdomains, and clathrin-containing-adhesion-complex [78]. For cell/cell interactions, hemidesmosome (HD) and TEMs are also involved in addition to integrins and AJ.

In Figure 2, we attempted to integrate different notions that have been described concerning the involvement of MLP during the mechanotransduction, considering the different levels of cell sensing. High mechanical stress leads to the recruitment of LIM-containing proteins [6,66,149]. Mechanosensing can be associated with the LIM domain and MLPs can play an important role in accommodating the balance between the exogenous and endogenous forces. Inside the cell, MLPs are found in costamer [129,130] sarcomeric Z-line [87], sarcomeric M-band [131], and intercalary disc [118,150]. As stated in the previous paragraph, the polymeric form of MLP has a structural function in the cytosketon, while its monomeric form has a signaling pathway activity upon post-translational modifications. The monomeric form becomes activated once it undergoes post-translational modifications [151]. These modifications include the acetylation which has been observed on the NLS fragment at the K69 position affecting calcium sensitivity [152], contractility in addition to shuttling into the nucleus, phosphorylation that can occur in several sites [122], sumoylation involved in MLP transcriptional activity [9], and gluthathionylation of C25 LM1 domain determined by proteomic studies in cardiomyocytes [153]. Notably, both the induction and functional consequence of these modifications are not well known and required more studies. It is tempting to propose that MLP could behave as observed for deformable teletonin and myosin, which expose their phosphorylation sites upon mechanical tensions leading to hypertrophy [6].

### 5.5. MLP Involvement in Human Diseases

This review aims to introduce the function of MLP in the mechanotransduction process and how by its dual activities (structural and cotranscription factor), it has a prominent role in the regulation of muscle homeostasis. The involvement of MLP in myopathy and muscle atrophy can be highlighted only by two recent reports. One report by Han et al. [154] on the *CSRP3−/−* chicken model showed a suppression of satellite cell differentiation through a defect in the TGF β/Smad3 signaling pathway. The second report performed by Chang et al. [155] on *CSRP3−/−* zebrafish resulted in myopathy in the form of cell damage and a decreased strength associated with decreased mechanosensing.

In contrast, the involvement of MLP in cardiomyopathies has been widely investigated. In the human gene mutation database, 47 missense/nonsense and splicing variants of CSRP3 have been registered (http://www.hgmd.cf.ac.uk (accessed on 1 July 2021)). These mutations have been associated with dilated cardiomyopathy (DCM) or hypertrophy cardiomyopathy (HCM) [156,157,158]. Moreover, different models have been generated by knocking out the CSPR3 gene [154,155,159]. Interestingly, the level of oligomeric MLP was found to be significantly reduced and, consequently, a concomitant increase in the monomeric MLP form in human failing hearts and various skeletal myopathies [89,123,160]. One of the main causes of hypertrophy and heart failure in these mutants is the alteration in MLP nucleocytoplasmic shuttling [10]. After prolonged mechanical overload, the mutated MLP is mislocalized leading to an impaired mechanosensing in cardiomyocytes that result in hypertrophy [159,160]. A recent study has elegantly described how vital it is to have a stabilized Z-disc MLP mechanosensory to prevent hypertrophic cardiomyopathy and heart failure [131]. Using derived cardiomyocytes from patient-specific-induced pluripotent stem cells, the authors have found that mutations on sarcomeric protein MYH7-R723C and MLP-W4R act synergistically to upregulate sarcomeric force production, destabilizing MLP leading to the disinhibition of calcineurin-NFAT (nuclear factor of activated T cells) signaling, remodeling, and hypertrophic responses. Rescuing the phenotype by gene editing or pharmacological treatments that inhibit NFAT have reduced the hypertrophy.

Overall, cells and tissues are continuously exposed to forces from the environment. They could be both endogenous forces (shear stresses caused by blood flow or respiration, and compression and tension forces from body movement) or exogenous ones from physical stimulation. It is of paramount importance to understand the activation of different mechanosensors molecules as MLP, which are able to activate specific signaling pathways leading to the alteration in mechanosensitive genes. Knowing the impact and conditions that the mechanical cues will contribute to every disease would be a source of possible therapeutic intervention in addition to restoring any altered gene expression.

## 6. Conclusions

To date, the data obtained have described MLP as a structural muscle protein localized in the submembrane compartment and in the cytoskeleton close to molecular cell motors. It is involved in mechanical interactions as a protein on a rope, and contributes to connecting pillar proteins during the transmission of forces from the extracellular matrix to the intracellular matrix as well as during the transmission of forces from sarcomeres to the cell membrane. When this protein is mechanically stressed, it undergoes a modification of its state from a polymeric form to a monomeric active form. This latter is involved in an anabolic process. Once translocated from the cytosol to the nucleus, it becomes a transcriptional cofactor, which is implied in a “direct” signaling pathway by mechanotransduction. Therefore, it could be hypothesized that repetitive membrane pressures/depressions may induce a mechanical stress on MLP, activating this protein and inducing an anabolic process. One could suggest that cycles of pressures/depressions of the muscular cell membrane as obtained by ultrasound stimulation would activate this signaling pathway. In the future, this would be a noninvasive way to counteract the muscle atrophy induced by anabolic versus catabolic disorders coming from the environment, aging or myopathies.
